# Novel Enzyme Actions for Sulphated Galactofucan Depolymerisation and a New Engineering Strategy for Molecular Stabilisation of Fucoidan Degrading Enzymes

**DOI:** 10.3390/md16110422

**Published:** 2018-11-01

**Authors:** Hang T. T. Cao, Maria D. Mikkelsen, Mateusz J. Lezyk, Ly M. Bui, Van T. T. Tran, Artem S. Silchenko, Mikhail I. Kusaykin, Thinh D. Pham, Bang H. Truong, Jesper Holck, Anne S. Meyer

**Affiliations:** 1 Protein Chemistry and Enzyme Technology, DTU Bioengineering, Department of Biotechnology and Biomedicine, Technical University of Denmark, Building 221, 2800 Kongens Lyngby, Denmark; caohang.nitra@gmail.com (H.T.T.C.); mdami@dtu.dk (M.D.M.); mateusz.lezyk@put.poznan.pl (M.J.L.); jesho@dtu.dk (J.H.); 2 NhaTrang Institute of Technology Research and Application, Vietnam Academy of Science and Technology, 02 Hung Vuong Street, Nhatrang 650000, Vietnam; bminhly.nitra@gmail.com (L.M.B.); vanvvlnt@yahoo.com.vn (V.T.T.T.); ducthinh.nitra@gmail.com (T.D.P.); truonghaibangnt@gmail.com (B.H.T.); 3 Laboratory of Enzyme Chemistry, G.B. Elyakov Pacific Institute of Bioorganic Chemistry, Far Eastern Branch, Russian Academy of Sciences, 159 100-Let Vladivostoku Ave., Vladivostok 690022, Russia; artem.silchencko@yandex.ru (A.S.S.); mik@piboc.dvo.ru (M.I.K.)

**Keywords:** fucoidan, endo-fucoidanase, galactofucan, molecular stabilisation, *Sargassum mcclurei*, *Turbinaria ornata*

## Abstract

Fucoidans from brown macroalgae have beneficial biomedical properties but their use as pharma products requires homogenous oligomeric products. In this study, the action of five recombinant microbial fucoidan degrading enzymes were evaluated on fucoidans from brown macroalgae: *Sargassum mcclurei*, *Fucus evanescens*, *Fucus vesiculosus*, *Turbinaria ornata*, *Saccharina cichorioides*, and *Undaria pinnatifida*. The enzymes included three endo-fucoidanases (EC 3.2.1.-GH 107), FcnA2, Fda1, and Fda2, and two unclassified endo-fucoglucuronomannan lyases, FdlA and FdlB. The oligosaccharide product profiles were assessed by carbohydrate-polyacrylamide gel electrophoresis and size exclusion chromatography. The recombinant enzymes FcnA2, Fda1, and Fda2 were unstable but were stabilised by truncation of the C-terminal end (removing up to 40% of the enzyme sequence). All five enzymes catalysed degradation of fucoidans containing α(1→4)-linked l-fucosyls. Fda2 also degraded *S. cichorioides* and *U. pinnatifida* fucoidans that have α(1→3)-linked l-fucosyls in their backbone. In the stabilised form, Fda1 also cleaved α(1→3) bonds. For the first time, we also show that several enzymes catalyse degradation of *S. mcclurei* galactofucan-fucoidan, known to contain α(1→4) and α(1→3) linked l-fucosyls and galactosyl-β(1→3) bonds in the backbone. These data enhance our understanding of fucoidan degrading enzymes and their substrate preferences and may assist development of enzyme-assisted production of defined fuco-oligosaccharides from fucoidan substrates.

## 1. Introduction

Fucoidan polysaccharides are a family of sulphated, fucose-rich polysaccharides uniquely produced by brown marine macroalgae (seaweeds) and certain marine invertebrates, such as sea cucumbers [1,2]. In general, fucoidans, also known as fucose-containing sulphated polysaccharides (FCSPs), consist of a backbone of α-L fucosyl residues linked together by (1→3) and/or (1→4)-glycoside bonds. The bonds are organised in stretches of α(1→3) or of alternating α(1→3)- and α(1→4)-glycoside linkages, depending on the macroalgal origin of the fucoidan, i.e., the species, age, geographical origin, and collection time (season) [3]. The l-fucosyl residues may be sulphated (−SO_3_^−^) at position C2 and/or C4 (rarely at C3). Some fucoidans have fucose, galactose, glucuronic acid or other mono- and oligosaccharides as short branches [1,4,5]. Galactofucans are the most structurally diverse group of fucoidans that have been characterised from brown algae to date. The galactofucans have galactose residues in their backbone or in their branches; the position and number of these galactose residues depend on the type of algae [6,7].

**Figure 1:**
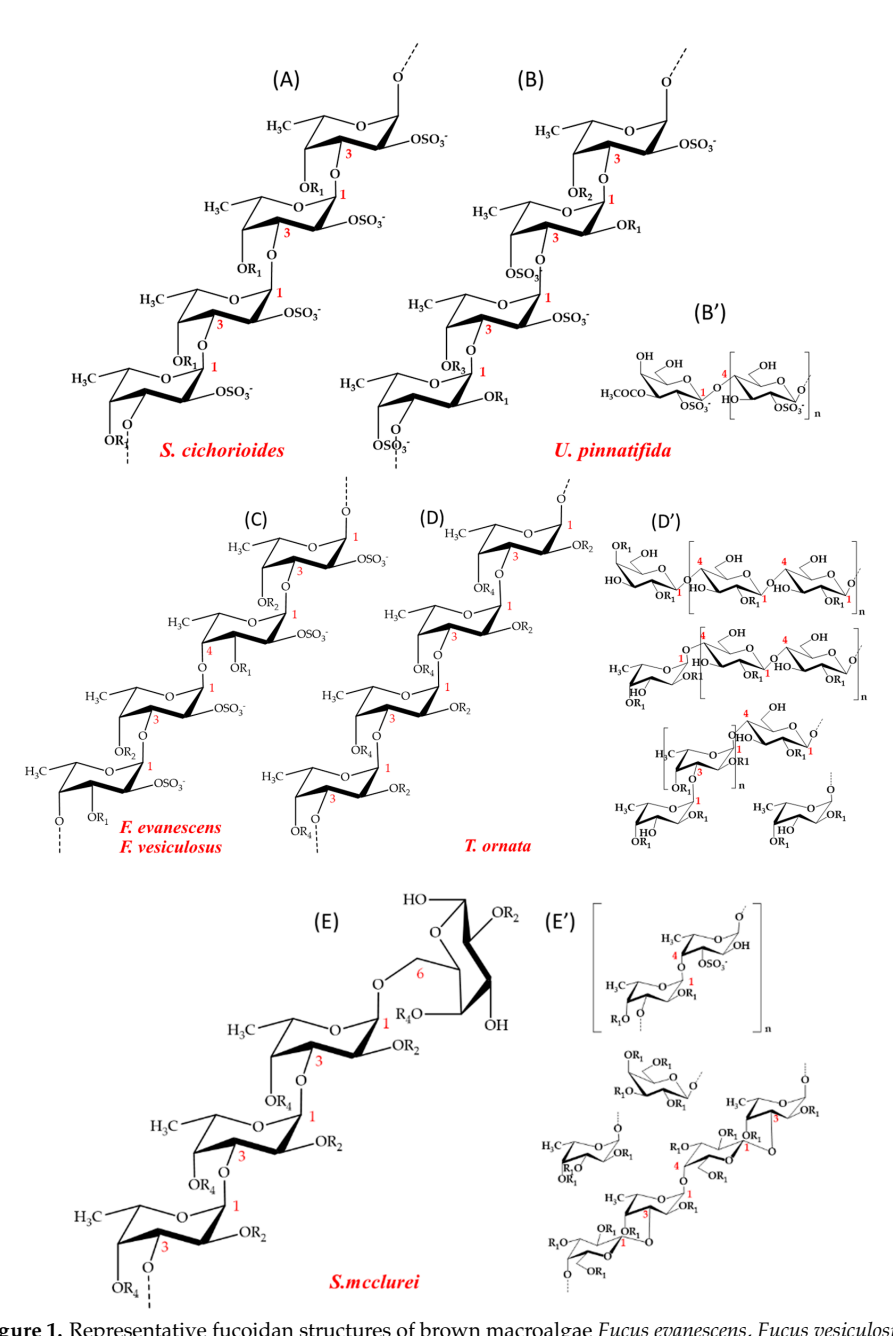
Representative fucoidan structures of brown macroalgae *Fucus evanescens*, *Fucus vesiculosus*, *Sargassum mcclurei*, *Turbinaria ornata*, *Saccharina cichorioides*, and *Undaria pinnatifida*: (**A**) main chain of *S. cichorioides* composed of α(1→3)-l-fucosyls; (**B**) main chain of *U. pinnatifida* fucoidan also composed of α(1→3)-l-fucosyls; (**B’**) branches of *U. pinnatifida* fucoidan [11]; (**C**) main chain of *F. evanescens* [1] and *F. vesiculosus* fucoidan [8,9,10], both composed of α(1→3)- and α(1→4)-linked l-fucosyls; (**D**) main chain of *T. ornata* fucoidan composed of α(1→3)-l-fucosyls [13,14]; (**D’**) branches of *T. ornata* of α(1→3)-l-fucosyls or of β(1→4)galactosyls and mixed fucosyl-galactosyls; (**E**) main chain of *S. mcclurei* fucoidan made up of mainly α(1→3)-l-fucosyls [12]; and (**E’**) branches or inserts in the main chain of *S. mcclurei* fucoidan. In all fucoidan structures: R_1_: −H or −SO_3_^−^; R_2_: −H, −SO_3_^−^ or H_3_COC−; R_3_: SO_3_^−^, H_3_COC− or branches; and R_4_: SO_3_^−^ or branches.

The structural diversity of fucoidans or FCSPs is very high as both the sulphatation pattern and the backbone bond pattern of α(1→3) and α(1→4)-glycosidic bonds vary significantly depending on the fucoidan source. The fucoidan from *Fucus vesiculosus*, which is available commercially, is known to be made up of a backbone of repeating disaccharide units of α(1→3)- and α(1→4)-linked sulphated l-fucosyl residues (C2, C2/C3, C2/C4, C4 sulphatation) [8,9,10] (Figure 1). Fucoidan from *Fucus evanescens* has a similar l-fucosyl backbone of alternating α(1→4) and α(1→3) l-fucosyls with sulphate substitution at C2. An additional sulphate may occupy position 4 in some of the α(1→3)-linked fucosyls, and the remaining hydroxyl groups may be randomly acetylated [1] (Figure 1). In contrast, the bonds in the backbone of the fucoidan from *Undaria pinnatifida* and *Saccharina cichorioides* are exclusively α(1→3). The backbone *U. pinnatifida* fucoidan is moreover assumed to be rich in 2,4-disulphate substituted fucosyl residues and to contain some β(1→4)-linked galactosyl residues as branches [11] (Figure 1). Some fucoidans have even more complex backbone structures as is the case, e.g., for fucoidan from the brown macroalgae *Sargassum mcclurei* and *Turbinaria ornata* commonly found along the Pacific Ocean coastline of Vietnam. The *S. mcclurei* fucoidan is essentially a sulphated galactofucan polysaccharide having both α(1→3) and α(1→4) linked fucosyl residues, as well as galactosyl-β(1→3) links to fucosyl, and α(1→6) linkages from fucosyl to galactosyl in the reducing end of the backbone (Figure 1). The fucosyl residues in *S. mcclurei* fucoidan are moreover differentially sulphated at C2 and/or at C4 and some of the galactosyl moieties are sulphated at C6 [12] (Figure 1). Fucoidan extracted from *T. ornata* collected at Nha-Trang bay, Vietnam, also seems to be a galactofucan. The backbone of *T. ornata* fucoidan has thus been proposed to consist of α(1→3)-linked l-fucosyls with galactosyl branches (Fuc:Gal ≈ 3:1) and has been found to have a high sulphate content of about 25% with sulphate attached mostly at C2, and to a lesser extent at C4, of both the fucosyl and the galactosyl residues [13,14] (Figure 1). The biological function of fucoidans in brown macroalgae is uncertain, but fucoidans have long been known to exert beneficial biological activities including anti-tumorigenic, immune-modulatory, anti-inflammatory, anti-coagulant and anti-thrombotic effects, as demonstrated in vitro and in vivo [14,15,16]. Fucoidan from *S. mcclurei*, including the unique galactofucan structural moieties with sulphated α(1→3) l-fucosyl and α(1→4) linked galactosyl residues, have for example been shown to inhibit colony formation of DLD-1 human colon cancer cells in vitro [12], and crude, sulphated fucoidan products extracted from *F. vesiculosus* and *Sargassum* spp. are known to cause growth inhibition and apoptosis of melanoma B16 cells in vitro and to enhance the activity of natural killer cells in vivo in mice resulting in the specific lysis of YAC-1 cells (a murine T-lymphoma cell line sensitive to natural killer cells) [15]. However, the high molecular weight, irregular structure, and viscosity of fucoidans are an obstacle for providing homogeneous preparations for soluble and concentrate pharmaceutical use. One approach to solve this problem is to use enzymes that can depolymerise the fucoidans providing a preparation that is easier to handle and also with potentially bioactive properties. 

About 20 microorganisms, mainly marine bacteria, have been described that produce fucoidanases [17,18,19,20,21]. In addition, a few fucoidanases have been found in marine molluscs [22,23]. In 2006, the gene encoding a fucoidanase from the marine bacterium *Mariniflexile fucanivorans* SW5T was cloned and the recombinant enzyme named FcnA. A C-terminal truncated version of FcnA named FcnA2 was previously reported to exert endo α(1→4) action on fucoidan from *Pelvetia canaliculata* (a type of fucoidan encompassing both α(1→4) and α(1→3) fucosyl-linkages in the backbone) [24]. In 2002, the genes encoding for two endo-fucoidanases referred to as Fda1 and Fda2, from the marine bacterium *Alteromonas* sp. SN-1009 were sequenced and their use for degradation of sulphated fucoidan originating from the brown seaweed *Kjellmaniella crassifolia* (now called *Saccharina sculpera*) were patented [25]. In the patent, these enzymes were reported to catalyse cleavage of α(1→3)-glycosidic bonds in the *K. crassifolia* (*S. sculpera*) fucoidan [25]. FcnA, Fda1, and Fda2 all belong to the new glycoside hydrolase family GH107 in CAZy [26]. In 2017, two endo-fucoidanases, FFA1 and FFA2, from the marine bacterium *Formosa algae* (KMM 3553T) were characterised and also suggested to belong to GH family 107 [27,28]. The FFA2 enzyme was proposed to be a poly[(1→4)-α-l-fucoside-2-sulphate] glycano hydrolase [27]. Already in 2003 Sakai et al. reported the finding of a new type of extracellular endo-fucoidan-lyase activity from “*Fucobacter marina*” SA-0082, or more correctly *Flavobacterium* sp. SA-0082, which acted on sulphated fucoglucurono-mannan from *K. crassifolia* (*S. sculpera*) [29,30]. By sequence analyses, it was found that this lyase activity was apparently encoded by two separate coding regions. Recombinant expression of these two putative fucoidan degrading enzymes, referred to as FdlA and FdlB, respectively, showed that the two enzymes had about 56% amino acid sequence identity and both were claimed to act as (glucurono-) fucoidan lyases on *K. crassifolia* (*S. sculpera*) fucoidan [25]. 

The objective of this work was to compare the catalytic properties, notably the substrate degradation patterns, on different fucoidans of the three GH107 endo-fucoidanases (EC 3.2.1.-) referred to as FcnA2, Fda1, and Fda2, and the two enzymes previously reported to be endo-fucoglucuronomannan-lyases, referred to as FdlA and FdlB. The action of the enzymes on different fucoidan substrate structures was compared by assessing oligomer product profiles resulting after treatment with recombinantly produced enzymes on fucoidans originating from six different types of brown seaweeds: *Sargassum mcclurei*, *Turbinaria ornata*, *Fucus evanescens*, *Fucus vesiculosus*, *Saccharina cichorioides*, and *Undaria pinnatifida*. We also report stabilisation of the recombinantly produced enzymes by targeted gene truncation resulting in deletion of large parts of the C-terminal end of several of the enzymes.

## 2. Results

### 2.1. Recombinant Enzyme Expression

**Figure 2:**
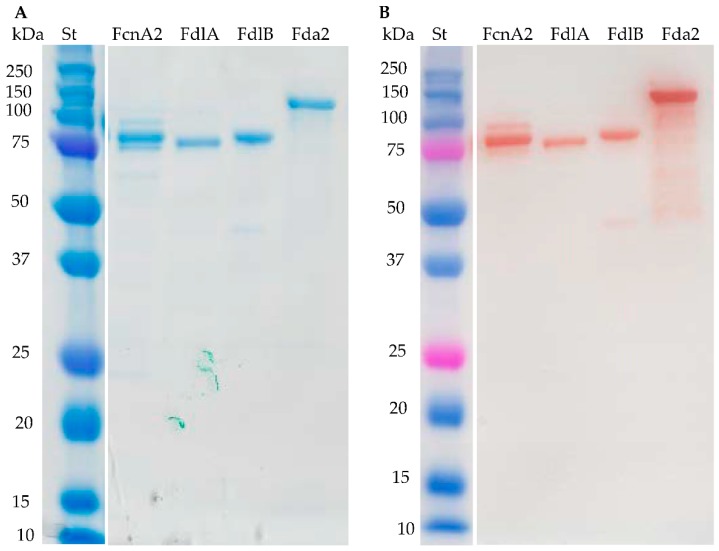
Purified recombinantly expressed fucoidan-modifying enzymes. (**A**) SDS-PAGE, and (**B**) Western blot of purified FcnA2, FdlA, FdlB, and Fda2. (St) is the protein plus molecular weight marker. The expected molecular weights of the recombinant enzymes FcnA2, FdlA, FdlB, and Fda2 were 87, 75, 76, and 94 kDa, respectively. The multiple bands seen for FcnA2 and Fda2, notably in the Western blot, indicate partial degradation of the proteins. Expression of recombinant Fda1 resulted in insoluble enzyme material which is not shown in this figure.

The enzymes FcnA2, FdlA and FdlB expressed well and the purified enzymes gave the expected band sizes as assessed by Sodium Dodecyl Sulfate Polyacrylamide Gel Electrophoresis (SDS-PAGE) (Figure 2A). The expression of recombinant Fda1 was high, but the protein remained in the cell debris after sonication (Figure S1). Several culture conditions for enzyme expression (temperature, medium, and isopropyl β-d-1-thiogalactopyranoside (IPTG) concentration) were tested to obtain a soluble enzyme, but without success. Fda2 expressed well, but migrated slower in the SDS-PAGE gel than expected (94 kDa). At present, the data do not allow any firm conclusions to be drawn regarding the cause of this retarded migration of the Fda2 protein, but high hydrophobicity and high levels of charged amino acids may cause anomalous SDS-PAGE migration as compared to the soluble, commercial protein standards [31]. For the enzymes FcnA2 and Fda2 more than one band was visible in both the SDS-PAGE gel and in the Western blot (Figure 2), suggesting spontaneous degradation rather than impurities from other proteins. This observation agrees with previously published data for recombinantly expressed FcnA2 reporting “co-elution” with other proteins, which could not be separated by anion exchange or SEC [24]. For the Fda2 enzyme, use of protease inhibitors such as PMSF (36978) from Thermo Fisher Scientific (Waltham, MA, USA) and a protease inhibitor cocktail (P8849) from Sigma-Aldrich (Steinheim, Germany) during purification did not improve stability, corroborating that the degradation likely occurred during expression in *E. coli* cells or during the subsequent purification.

### 2.2. Substrate Specificity of the Recombinant Fucoidan-Degrading Enzymes

**Figure 3:**
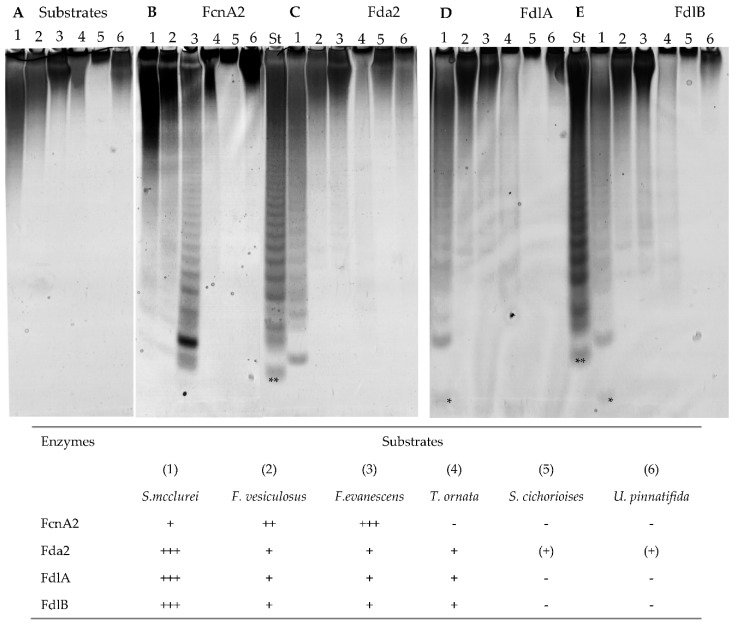
C-PAGE analysis of fucoidan degradation using purified enzymes: (**A**) substrate control with no enzyme; and (**B**–**E**) enzymatic products from reaction of FcnA2, Fda2, FdlA, and FdlB on different fucoidans, respectively (expression of recombinant Fda1 resulted in insoluble enzymes, which is why there are no data for Fda1): (1) *Sargassum mcclurei*; (2) *Fucus vesiculosus*; (3) *Fucus evanescens*; (4) *Turbinaria ornata*; (5) *Saccharina cichorioides*; and (6) *Undaria pinnatifida*. The extent of degradation is indicated with: (+++) highest, (++) medium, (+) lowest and (+) is positive activity resulting in a high molecular smear, while (−) is no activity. The standard (St) is the product profile of FFA2 treatment of fucoidan from *F. evanescens*. The lowest band (**) of the St is a tetra-saccharide of (1→4)- and (1→3)-linked α-l-fucosyls sulphated at every C2 with an approximate mass of 972 Da [27]. (*) indicates an enzymatic fucoidan degradation product of either lower mass or higher charge than the lowest St band (**) compound. The reaction time was 24 h.

The six different fucoidan samples were treated with the purified enzymes FcnA2, Fda2, FdlA, and FdlB and the treatments produced different carbohydrate-polyacrylamide gel electrophoresis (C-PAGE) patterns with the six fucoidan samples (the expression of recombinant Fda1 resulted in insoluble enzymes, which is why there are no data for Fda1). The reactions were run for 24 h to ascertain maximal substrate degradation. Preliminary data using higher enzyme dosage or longer reaction time did not show higher extent of degradation except of the *S. mcclurei* fucoidan that gave more visible bands in the C-PAGE after a 48 h reaction (these data are discussed further below). Hence, the data obtained by C-PAGE showed both the selectivity and the maximal extent of fucoidan degradation obtainable for each set of enzyme and substrate. This means that it is presumed that the unreacted higher molecular weight polysaccharides do not contain structural units, i.e., backbone-stretches, linkages, substitutions or branches, attackable by the particular enzyme examined. The positive control standard (St) was the hydrolysate from the enzymatic reaction of the *Formosa algae* FFA2 on *F. evanescens* fucoidan, where the lowest band corresponds to a tetra-saccharide of (1→4)- and (1→3)-linked α-l-fucosyls with each fucosyl residue sulphated at C2 [27] (Figure 3). The data obtained by C-PAGE indicated more extensive degradation of the fucoidan substrates from *Sargassum mcclurei* (1), *Fucus vesiculosus* (2), and *Fucus evanescens* (3) than of substrates predominantly having α(1→3) glycoside bonds in their backbone structures, originating from *Turbinaria ornate* (4), *Saccharina cichorioides* (5), and *Undaria pinnatifida* (6), respectively (Figure 3). In general, the data obtained show that each enzyme produced differently sized sulphated oligomers in the C-PAGE chromatograms, suggesting that the different enzymes target different linkages and/or differently sulphated fucosyl residues. The results also suggest that all the enzymes were endo-acting as the enzymatic action left behind relatively high molecular weight fractions.

### 2.3. FcnA2 Catalyses Cleavage of α(1→4) Fucosyl Bonds in Sulphated Fucoidan Backbones

The recombinantly expressed FcnA2 enzyme exerted highest activity on the fucoidan from *F. evanescens*, and the degradation of this substrate was much more profound than on *F. vesiculosus*, even though both substrates have similar alternating α(1→3) and α(1→4) glycoside bonds in the backbone. The degradation of fucoidan from *F. evanescens* was in agreement with previous data showing that FcnA2 is able to degrade fucoidan from *Pelvetia canaliculata* [24]. The fucoidans from *F. evanescens* and *P. canaliculata* presumably have less if any C2, C4 disulphates in the “−1” position of the α(1→4)-l-fucosyl linkage compared to the fucoidan substrate from *F. vesiculosus*, which likely contains more fucosyl residues with C2/C4 and even some with C2/C3 disulphatation than the *F. evanescens* fucoidan. The lesser degree of C2/C4 and C2/C3 disulphatation might be the reason for the *F. evanescens* fucoidan being more degraded than the *F. vesiculosus* fucoidan (Figure 3). Hence, FcnA2 most likely catalyses cleavage of (1→4)-α-glycosidic bonds between the −1 fucosyl residues having the sulphate group at C2, but not at both C2, C4. However, detailed structural elucidation of the fucoidan products and modelling of the substrate accommodation in the enzyme’s active site are warranted to substantiate this hypothesis. The differences in the degradation of fucoidan from *F. evanescens* and *F. vesiculosus* thus indicate that differences in the sulphatation pattern or in other types of substitutions on the substrate backbones may influence the action of FcnA2 on these two *Fucus* sp. derived fucoidans. The data suggest that the presumed presence in *F. vesiculosus* of fucosyl residues with disulphate at C2, C4 (on either the −1 or +1 position of the α(1→4) glycoside bond) may retard the enzymatic action of FcnA2. 

The smallest oligomers released from *F. evanescens* by FcnA2 also differed from those released by the FFA2 treatment of fucoidan from *F. evanescens* in the standard (st) (Figure 3). FFA2 catalyses the cleavage of (1→4)-α-glycosidic bonds in the *F. evanescens* fucoidan within the structural fragment [→3)-α-l-Fucp2S-(1→4)-α-l-Fucp2S-(1→]n but not in the fragment [→3)-α-l-Fucp2S,4S-(1→4)-α-l-Fucp2S-(1→]n. The difference in the oligomers released suggests that the sulphatation preferences of the FFA2 and FcnA2 may differ, which invites to further elucidation of the enzyme structures and detailed analyses and modelling of enzyme-substrate interactions. FcnA2 also catalysed degradation of the sulphated galacto-fucan fucoidan from *S. mcclurei* resulting in production of several low molecular weight bands in the C-PAGE (Figure 3B). The partial degradation is in agreement with the enzyme attacking α(1→4) linked (sulphated) l-fucosyl residues. Nevertheless, this enzymatic degradation of *S. mcclurei* fucoidan is a novel finding, as enzymatic modification of the *S. mcclurei* fucoidan has not previously been reported. The apparent lack of action of FcnA2 on the fucoidan from *T. ornata*, *S. cichorioides*, and *U. pinnatifida* suggests that FcnA2 does not catalyse cleavage of α(1→3) bonds between fucosyl residues, whereas the activity on the other three substrates supports the hypothesis that the enzyme attacks α(1→4) bonds between l-fucosyl residues as previously shown [24].

### 2.4. Fda2 Catalyses Cleavage of α(1→3) Fucosyl Bonds in Sulphated Fucoidan Backbones

Fda2 catalysed partial degradation of the galactofucan-rich fucoidan from *S. mcclurei* similar to the action of FcnA2 (Figure 3C). The C-PAGE results showed that this enzyme also exerted partial degradation of the fucoidans from *F. vesiculosus* and *F. evanescens* and had very low activity on the fucoidans rich in α(1→3) fucosyl linkages from *T. ornata*, *S. cichorioides*, and *U. pinnatifida*. The activity was very low, but still visible on the *S. cichorioides* fucoidan (with a smear at the top of the gel and weak bands in the lower part of the gel) and on the *U. pinnatifida* fucoidan (with a discernible smear at the top of the gel) (Figure 2C). The action of Fda2 on *S. mcclurei* fucoidan is a new finding which suggests that the Fda2 enzyme may be employed for controlled degradation of the complex galacto-fucan fucoidan from *S. mcclurei*. The activity of this enzyme on *S. mcclurei*, *F. evanescens* and *F. vesiculosus* together with the weak activity observed on substrates rich in α(1→3) fucosyl linkages corroborates previous claims of the action of Fda2 on α(1→3) bonded l-fucosyls in fucoidan [25]. Both Fda1 and Fda2 were previously shown to digest sulphated fucans from *K. crassifolia* (i.e., *S. sculpera*) with the backbone structure [3)-α-l-Fucp-(2OSO_3_)-1→3-α-l-Fucp-(2,4OSO_3_)-(1→] and to partially digest fucoidan from other brown algae of the order Laminariales, such as *Saccharina japonica*, *Lessonia nigrescens*, and *Ecklonia maxima* [32]. The data obtained further support the hypothesis that Fda2, despite its instability (Figure 2B), catalyses cleavage of α(1→3) fucosyl bonds in sulphated fucoidan backbones. 

### 2.5. FdlA and FdlB Action

The FdlA and FdlB enzymes originating from *Flavobacterium* sp. SA-0081 (previously referred to as “*Fucobacter marina*”) (Table 1) have been claimed to be specific for certain sulphated fuco-glucuronomannan (SFGM) structural fragments containing uronic acid and d-mannosyl α-linkages in fucoidan molecules [25]. The enzymes were purified from the SA-0082 strain and were shown to catalyse cleavage of SFGM fractions from the brown algae *Kjellmaniella crassifolia* (now *S. sculpera*) via a lyase mechanism cleaving the α-linkage between d-mannosyl and d-glucuronate in the SFGM fractions [33].

In this study, FdlA and FdlB both exerted activity on the fucoidans from *S. mcclurei*, *F. vesiculosus*, and *F. evanescens.* Only weak action was observed on fucoidans from *T. ornata* and essentially no activity on *S. cichorioides* and *U. pinnatifida* was found (Figure 3D,E). Fucoidan preparations from *S. mcclurei*, *T. ornata*, *F. evanescens* and *F. vesiculosus* may contain low amounts of uronic acid and sometimes traces of mannose [12,14,33,34,35] but until now no data show that d-mannosyl and d-glucuronate are present in the backbone of these fucoidans. Moreover, no lyase activity was detected by monitoring absorbance at 232 nm, indicating that the degradation products did not include unsaturated uronic oligosaccharides. FdlA and FdlB most likely cleave α(1→4) fucosyl bonds in the backbone of these fucoidans, since lack of activity on fucoidan from *S. cichorioides* and from *U. pinnatifida* (and weak action on *T. ornata* fucoidan) indicate that FdlA and FdlB do not cleave the α(1→3) bonds in fucoidan. 

The similar weak degradation of the fucoidan substrates from *F. vesiculosus* and *F. evanescens* by both enzymes, i.e., producing almost similar oligomer profiles in the C-PAGE, suggests a preference for rare or complex fucosyl-sulphatation (e.g., C2 and C4) in the *Fucus* fucoidan substrates. Such substrates may occur more abundantly in *S. mcclurei* fucoidan (Figure 2), and the enzyme most likely prefers to attack only α(1→4) fucosyl-bonds. Enzyme FdlB appeared to exert a more profound action on the *F. evanescens* substrate than FdlA. Interestingly, the action of the two enzymes on *S. mcclurei* galacto-fucan substrate produced a band that travelled further in the gel than the sulphated tetra-saccharide of the control, suggesting that both FdlA and FdlB are able to catalyse disintegration of sulphated fucoidan oligomers. Due to the high degree of depolymerisation, down to oligosaccharides of less than DP4 (Figure 3), and due to the high abundance of galactosyl residues in *S. mcclurei* fucoidan, we cannot rule out that FdlA and FdlB may cleave galactosyl-α(1→4) bonds (Figure 3D,E), and further analysis could confirm this conclusion.

### 2.6. Further Assessment of Sargassum mcclurei Fucoidan Degradation

**Figure 4:**
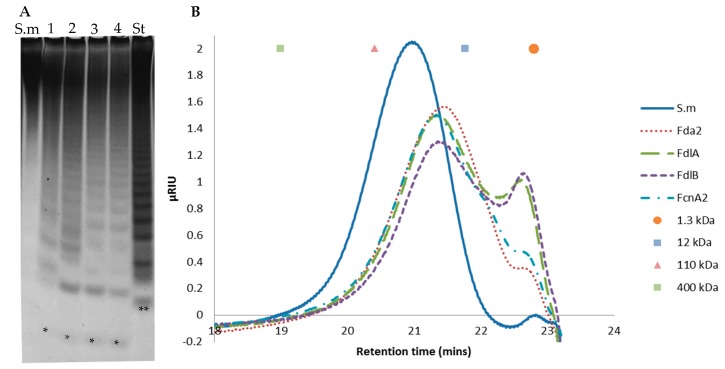
Degradation of *Sargassum mcclurei* fucoidan (S.m) by fucoidanase enzymes. (**A**) C-PAGE; and (**B**) size exclusion chromatography (SEC) of the products produced by: (1) FcnA2; (2) Fda2; (3) FdlA; and (4) FdlB on *S. mcclurei* fucoidan and molecular weight standards. The lowest band (**) of the standard (St), resulting from FFA2 treatment of fucoidan from *F. evanescens*, corresponds to a tetra-saccharide of (1→4)- and (1→3)-linked α-l-fucosyls with each fucosyl residue sulphated at C2; total mass has been calculated to be approximately 972 Da [27]. Reaction time was 48 h. (*) indicates an enzymatic fucoidan degradation product of either lower mass or higher charge than the lowest St band (**) compound.

C-PAGE and SEC of oligosaccharides released by the enzymes FcnA2, Fda2, FdlB and FdlA after extended reaction for 48 h, showed that each enzyme catalysed profound degradation of *S. mcclurei* fucoidan (Figure 4). 

In all cases, the smallest oligosaccharide ran further than the lowest of the standard, suggesting that the released oligosaccharides are either smaller or more charged, i.e., more sulphated, than the tetra-saccharide in the standard. The SEC profiles of the FcnA2 and Fda2 were similar, but the product profile differed from those of FdlA and FdlB which contained a smaller peak at around 22.5 min (corresponding to a molecular weight SEC standard of around 1.3 kDa), indicating that they acted slower if at all on certain fucoidan fragments <1.3 kD. Taken together with the C-PAGE results (Figure 4A), these data suggest that FdlA and FdlB exerted similar substrate attack preferences and left behind some oligomers around 1.3 kDa, whereas FcnA2 and Fda2 appeared to degrade the lower molar weight oligomers to a more significant extent.

### 2.7. New Construct of FcnA2

**Figure 5:**
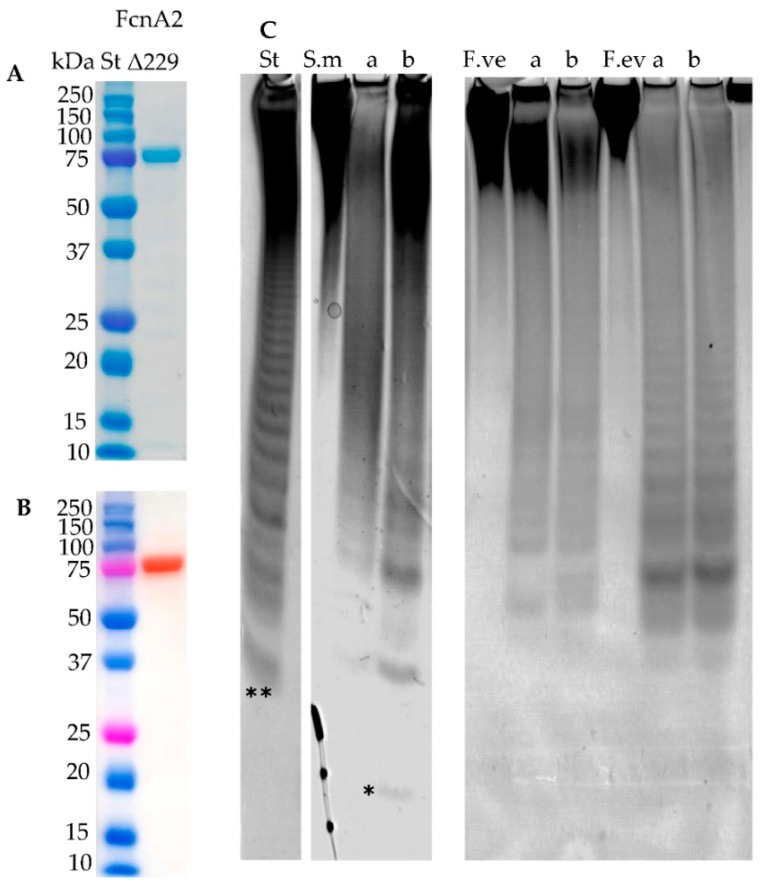
Purification and activity of enzyme FcnAΔ229. (**A**) SDS-PAGE indicating the expected molecular weight of 80 kDa and purity; (**B**) Western blot of purified FcnAΔ229. (St) is the protein plus molecular weight marker; and (**C**) enzyme activity by C-PAGE of (a) FcnA2 and (b) FcnAΔ229 on fucoidans from *S. mcclurei*, *F. vesiculosus* and *F. evanescens*. FcnA2 and FcnAΔ229 have similar profiles on *F. vesiculosus* and *F. evanescens* fucoidans. The reaction time was 24 h. The lowest band (**) of the standard (St), resulting from FFA2 treatment of fucoidan from *F. evanescens*, corresponds to a tetra-saccharide of (1→4)- and (1→3)-linked α-l-fucosyls with each fucosyl residue sulphated at C2; total mass has been calculated to be approximate 972 Da [27]. (*) An oligosaccharide of lower molecular weight or higher charge than the lowest band in the standard (**).

Western blot analysis of FcnA2 (Figure 2B) indicated that the spontaneous degradation of FcnA2 occurred from the C-terminal end, since the N-terminal His-tag was still present, making the protein visible in the Western blot. To avoid this degradation, a further truncation was made by removing an additional 80 amino acids from C-terminal end of the FcnA2 protein. This truncated enzyme was thus 229 amino acids shorter than the original FcnA enzyme and was called FcnAΔ229 (Table 1). FcnAΔ229 could be expressed very well and was purified with no apparent protein degradation, as illustrated by SDS-PAGE and Western blot analysis, giving the expected band size of 80 kDa (Figure 5A,B). FcnAΔ229 showed activity on the same substrates as FcnA2 (Figure 5C). 

However, an oligosaccharide was released after 24 h that was running further than what was observed for FcnA2 (Figure 5C). This result indicated that the change in stability conferred by deletion of the 80 amino acids in FcnA2 in turn apparently enhanced substrate degradation, but the truncation did not confer any other apparent changes in the *S. mcclurei* degradation profile.

### 2.8. Stabilisation through C-Terminal Truncation of Fda1 and Fda2

The expression of Fda1 was high but the protein remained in the cell debris after sonication (Figure S1). By sequence analyses of Fda1 and Fda2, it was found that both enzymes contained two predicted Laminin G domains (IPR001791) (LamG domains) towards the C-terminal of each protein (Figure S2). Western blot analysis of Fda2 (Figure 2B) also indicated that enzyme destabilisation occurred via degradation from the C-terminal end as was observed for FcnA2. Hence, a strategy to stabilise the enzymes by deletion of the two predicted LamG domains in Fda1 and in Fda2 was developed and new constructs of Fda1, called Fda1Δ145 (one LamG domain deleted) and Fda1Δ395 (both LamG domains deleted), were prepared (Table 1). An additional his-tag was included with these new constructs to ensure better binding to the Ni^2+^ Sepharose column. Notably for Fda2, in addition to being highly unstable, substantial amounts of protein were lost during purification, presumably due to lack of binding to the column (data not shown). This new construct was called Fda2-His (Table 1). In addition, as for Fda1, new constructs devoid of either one or both of the two predicted LamG domains of Fda2 were also constructed. These Fda2 C-terminal deletion mutants were named Fda2Δ146 and Fda2Δ390 (Table S1 and Figure S2). 

**Figure 6:**
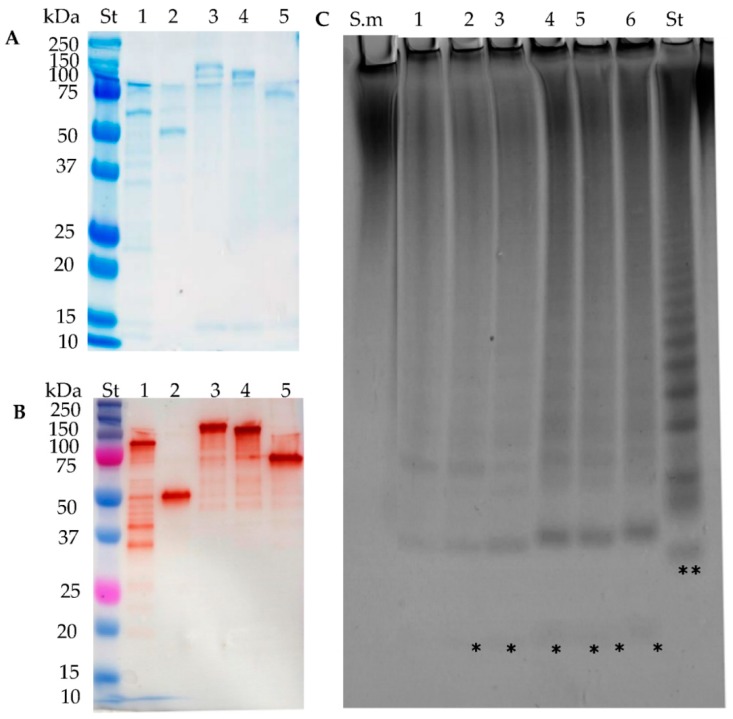
Purification and activity of Fda1 and Fda2 deletion mutants. (**A**) SDS-PAGE; and (**B**) Western blot of purified: (1) Fda1Δ145; (2) Fda1Δ395; (3) Fda2-C-His; (4) Fda2Δ146; and (5) Fda2Δ390. (St) is the protein plus molecular weight marker. The expected sizes of the proteins were 90, 50, 125, 110, 70 kDa respectively. (**C**) Enzymatic *S. mcclurei* fucoidan (S.m) degradation by C-PAGE: (1) Fda1Δ145; (2) Fda1Δ395; (3) Fda2-His; (4) Fda2Δ146; (5) Fda2Δ390; (6) Fda2; and the standard (St) resulting from FFA2 treatment of fucoidan from *F. evanescens*. The lowest band (**) of the standard (St), resulting from FFA2 treatment of fucoidan from *F. evanescens*, corresponds to a tetra-saccharide of (1→4)- and (1→3)-linked α-l-fucosyls with each fucosyl residue sulphated at C2; total mass has been calculated to be 972 Da [27]. (*) indicates an enzymatic fucoidan degradation product of either lower mass or higher charge than the lowest St band (**) compound. The reaction time was 48 h.

SDS-PAGE and Western blot analysis showed that all modified enzyme constructs expressed well. Some protein degradation was evident, but notably the double LamG deletion constructs, Fda1Δ395 and Fda2Δ390, appeared more stable than full length enzymes (Figure 6A,B). All the truncated enzymes exerted activity on *S. mcclurei* fucoidan, verifying the enzyme stabilisation strategy by LamG deletion (Figure 6C). Further study verified that Fda1Δ395 was stable but that degradation of the other truncated enzymes (Fda1Δ145, Fda2-C-His, Fda2Δ146, and Fda2Δ390) occurred already inside the *E. coli* cells, presumably via action of proteases in *E. coli*, recognising sites in the C-terminal of the enzymes, since degradation was evident in the *E. coli* cells before sonication (Figure S3).

### 2.9. C-Terminally Truncated Fda1 Attacks α(1→3)-Linkages

**Figure 7:**
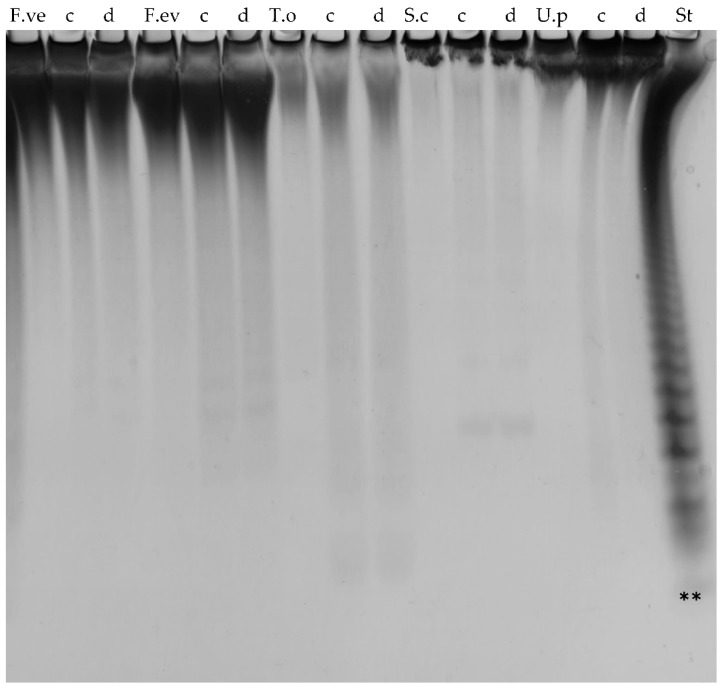
Enzyme activity of truncated Fda1 mutants by C-PAGE. Enzyme activity of (c) Fda1Δ145 and (d) Fda1Δ395 on fucoidans from *F. vesiculosus* (F.ve), *F. evanescens* (F.ev), *T. ornata* (T.o), *S. cichorioides* (S.c) and *U. pinnatifida* (U.p), and standard (st). Both enzymes show activity on all the tested substrates to a comparable degree. The lowest band (**) of the standard (St), resulting from FFA2 treatment of fucoidan from *F. evanescens*, corresponds to a tetra-saccharide of (1→4)- and (1→3)-linked α-l-fucosyls with each fucosyl residue sulphated at C2; total mass has been calculated to be 972 Da [27].

The truncated Fda1 proteins Fda1Δ145 and Fda1Δ395 both catalysed degradation of most of the fucoidan substrates, although compared to the degradation achieved on the *S. mcclurei* fucoidan, the extent of degradation appeared to be lower (Figure 7). Both truncated enzymes produced comparable degradation patterns, releasing fucoidan oligo-saccharides that migrated to the same extent within the C-PAGE gels. Interestingly, Fda1 mutants were able to catalyse the degradation of fucoidans rich in α(1→3) fucosyl linkages from *T. ornata*, *S. cichorioides* and *U. pinnatifida* (Figure 7), indicating that the C-terminally truncated Fda1 enzymes attack α(1→3)-linkages as previously described [25]. Removal of up to 47% of the Fda1 sequence from the C-terminal thus resulted in a more stable enzyme that retain activity.

## 3. Discussion

This work showed that different microbially derived fucoidan-degrading enzymes exert activity on an array of different fucoidan substrates from brown macroalgae, even the very complex *S. mcclurei* fucoidan. FcnA2, Fda2, FdlA, and FdlB were found to degrade *S. mcclurei* fucoidan, with Fda2, FdlA and FdlB having particularly high activity on this fucoidan, which is known to contain sulphated galacto-fucan structural units and both α(1→4) and α(1→3) l-fucosyl linkages (Figure 1). FcnA2 and FcnA2Δ229 were more active than all the other enzymes on fucoidan from *F. evanescens* and they were also more active on fucoidan from *F. evanescens* than on fucoidan from *F. vesiculosus* suggesting an effect of the substrate sulphatation pattern or of other structural features of the substrate. Fda2 was the only enzyme that degraded fucoidans rich in α(1→3) l-fucosyl linkages, but FdlA and FdlB were also able to at least partially degrade the fucoidan from *T. ornata*. FdlA and FdlB were previously claimed to be lyases acting on manno-glucurono-linkages in fucoidan from *K. crassifolia* (i.e., *S. sculpera*). In the present work these enzymes were specifically found to act as endo-fucoidanases on fucoidans devoid of these types of bonds and did not produce any unsaturated 4–5 oligosaccharide uronides.

Enzyme stabilization was successfully achieved by targeted truncation of the C-terminal ends of FcnA2, Fda1 and Fda2. Interestingly, for FcnA2, the stabilisation by C-terminal truncation, to the enzyme variant FcnAΔ229, resulted in an enzyme which appeared able to foster more profound degradation of the *S. mcclurei* fucoidan than the parent enzyme. For Fda1 and Fda2, successful expression and stabilisation were attained by LamG domain deletion, in turn this stabilisation allowed us to show the ability of the otherwise unstable Fda1 to catalyse degradation of the *S. mcclurei* fucoidan. The data obtained have implications for use of these enzymes, including the stabilised versions, in fucoidan processing.

Enzymatically produced short sulphated fuco-oligosaccharides, with degree of polymerisation of 4–10, derived from *Sargassum horneri*, obtained via treatment with a recombinant GH family 107 endo-fucoidanase, FFA1 (originating from the marine bacterium *Formosa algae*), were recently reported unable to suppress growth of DLD-1 human colon cancer cells in vitro, whilst this ability, i.e., potential anti-cancerogenic activity, is significant for native fucoidan from *S. horneri* [28]. In contrast, enzymatically produced sulphated fucoidan products from *F. evanescens* have been reported to have a better effect than the corresponding native, higher molecular weight fucoidan, on the functional activity of innate immunity cells in vitro [36]. Partially depolymerised fucoidan fractions from *Saccharina cichorioides* exert strong inhibition of colony formation of colorectal carcinoma cells HT-29 in vitro [37]. It is not yet known whether specific structural units of fucoidan backbones or if particular sidechains or substitutions on fucoidans confer specific bioactivity functions. The results of the present work enable targeted production of defined fucoidan oligomer products. The availability of such homogenous fucoidan oligomers will permit rigorous research studies on the putative pharmaceutical functions of fucoidans of different structural configurations. 

## 4. Materials and Methods

### 4.1. Fucoidan Substrates

Crude fucoidans from *Sargassum mcclurei*, *Fucus evanescens*, *Undaria pinnatifida*, and *Saccharina cichorioides* were extracted as described by Zvyagintseva et al. (1999) [38]. Fucoidan from *S. mcclurei* was purified further by ion-exchange chromatography [12]. *Turbinaria ornata* fucoidan was extracted as described by Thanh et al. (2013) [13]. Fucoidans from *F. evanescens*, *U. pinnatifida*, and *S. cichorioides* were purified as described by Kusaykin et al. (2006) [39]. Pure fucoidan from *Fucus vesiculosus* (F8190) was from Sigma-Aldrich (Steinheim, Germany).

### 4.2. Enzymes and Gene Constructs

Amino acid sequences for the five enzymes FcnA2, the C-terminal truncated version of FcnA (CAI47003.1) from *Mariniflexile fucanivorans* SW5 [24]; Fda1 (AAO00508.1) and Fda2 (AAO00509.1) from *Alteromonas* sp. SN-1009 (); FdlA (AAO00510.1) and FdlB (AAO00511.1) from *Flavobacterium* sp. SA-0082, were retrieved from GenBank (Table 1). The construct containing the gene encoding FcnA2 was designed to harbour an N-terminal 6xhistidine tag, while the gene constructs Fda1, Fda2, FdlA, and FdlB encoding the Fda1, Fda2, FdlA, FdlB proteins, respectively, were designed to harbour an N-terminal 10× histidine tag. The synthetic codon-optimised genes (optimised for *E. coli* expression), all devoid of their original signal peptide, were synthesised by GenScript (Piscataway, NJ, USA) and delivered as inserted either into the pET-45b(+) vector between the KpnI and PacI restriction sites (FcnA2) or into the pET-19b(+) plasmid vector between the NcoI and XhoI restriction sites (all other enzymes).

For FcnA2 C-terminal deletion of 80 amino acids of the enzyme equivalent to deletion of 229 amino acids of FcnA (GenBank No. CAI47003.1) was constructed, and the truncated protein was named FcnAΔ229 (Table 1). 

Both Fda1 and Fda2 contain two predicted laminin G (LamG) domains in the sequence. Deletion mutants devoid of one or both predicted LamG domains were constructed by PCR amplification of the codon-optimised genes, each with an additional C-terminal 10× histidine tag, using CloneAmp HiFi polymerase premix (Takara Bio USA Inc., Mountain View, CA, USA) (primer sequences are listed in Table S1). For Fda1, the truncated proteins were named Fda1Δ145 and Fda1Δ395, as 145 and 395 amino acids had been removed from the C-terminal end, respectively. Analogously, for Fda2, the truncated versions were named Fda2Δ146 and Fda2Δ390, indicating that 146 and 390 amino acids, respectively, had been removed from the C-terminal. The construct of Fda2-His was done by adding 10× histidine tag at the C-terminal end. After PCR amplification, products were digested with BsaI and XhoI and ligated into the pET19b (+) vector between the NcoI and XhoI sites. Positive clones were confirmed by DNA sequencing.

The *Escherichia coli* strain DH5α (Invitrogen^®^ Life Technologies, Thermo Fisher Scientific, Waltham, MA, USA), was used as plasmid propagation host. BL21 (DE3) and C41 (DE3) (also from Invitrogen^®^ Life Technologies) were used as expression hosts for the fucoidan-degrading enzymes (Table 1). Protein expression was done as described below. 

### 4.3. Production of Recombinant Enzymes

Expression of FcnA2 and FcnAΔ229 was performed in *E. coli* (BL21 (DE3) harbouring the Pch2 (pGro7) plasmid. Overnight cultures grown at 37 °C with agitation (180 rpm) in lysogeny broth (LB) medium containing 100 μg mL^−1^ ampicillin and 34 μg mL^−1^ chloramphenicol were used to inoculate 500 mL LB containing 100 μg mL^−1^ ampicillin, 34 μg mL^−1^ chloramphenicol and 0.05% arabinose. The inoculated LB was incubated at 37 °C with 180 rpm shaking until cultures reached 0.6-0.8 OD_600_. Enzyme expression was induced with 1mM IPTG for 20 h at 20 °C and 180 rpm.

Expression of Fda1, Fda1Δ145, Fda1Δ395, Fda2, Fda2-His, Fda2Δ146, and Fda2Δ390 was also performed in *E. coli* (BL21 (DE3) with Pch2 (pGro7)). Overnight cultures grown at 37 °C and 180 rpm in LB medium containing 100 μg mL^−1^ ampicillin and 34 μg mL^−1^ chloramphenicol were used to inoculate 500 mL auto-induction media containing 0.6% Na_2_HPO_4_, 0.3% KH_2_PO_4_, 2%, tryptone, 5% yeast extract, 5% NaCl, 0.6% glycerol, 0.05% glucose, 0.2% lactose, 0.05% arabinose, 100 μg mL^−1^ ampicillin and 34 μg mL^−1^ chloramphenicol. Cells were grown at 20 °C, 180 rpm for 20 h. Expression of FdlA, FdlB was performed in *E. coli* (C41 (DE3)). Overnight cultures grown at 37 °C and 180 rpm in LB medium containing 100 μg mL^−1^ ampicillin were used to inoculate 500 mL LB containing 100 μg mL^−1^ ampicillin and were grown at 37 °C and 180 rpm until cultures reached 0.6-0.8 OD_600_. The expression of the recombinant fucoidanases was induced with 1 mM IPTG during cell growth for 20 h at 20 °C and 180 rpm.

Cells were harvested by centrifugation at 5000× *g* for 20 min and 4 °C and the pellet was re-suspended in binding buffer (20 mM Tris-HCl buffer, 250 mM NaCl, 20 mM imidazole, pH 7.5) before being disrupted by UP400S Ultrasonic processor (Hielscher, Teltow, Germany)with 0.5 cycle and 100% amplitude. Cell debris was pelleted by centrifugation (20,000× *g*, 20 min at 4 °C). The supernatant obtained by centrifugation was then filtered through a 0.45 μm filter and applied to a 5 mL Ni^2+^ Sepharose HisTrap HP column (GE Healthcare, Uppsala, Sweden) which was equilibrated with binding buffer using an Äkta purifier (GE Healthcare, Uppsala, Sweden). The resin was washed 3 times with 20 mM Tris-HCl buffer, 250 mM NaCl, and 20 mM imidazole at pH 7.5 and proteins were eluted by a linear gradient of elution buffer (20 mM Tris-HCl buffer, 250 mM NaCl, and 20–500 mM imidazole, pH 7.5). The eluted fractions were analysed by sodium dodecyl sulphate–polyacrylamide gel electrophoresis (SDS-PAGE) and Western blotting as described below to assess the purity and homogenous fractions were pooled. Protein content was determined by the Bradford assay [40] with bovine serum albumin as standard.

### 4.4. SDS-PAGE

The homogeneity and molecular weight of the recombinantly expressed proteins were estimated by (SDS–PAGE) electrophoresis according to the Laemmli protocol [41]. Electrophoresis was performed in 12% acrylamide gels with the addition of 4× Laemmli loading-buffer, to 40 µg of crude protein and 5 µg purified protein and 5 mM DTT. The analysis of total intracellular proteins was conducted by using the biomass from 300 µL culture with 100 µL of 4× Laemmli loading-buffer, 10 µL of samples were loaded on the 12% acrylamide gels. The Protein Plus molecular weight marker (Bio-Rad Laboratories, Hercules, CA, USA) with molecular weights of 10–250 kDa was used as standard. 

### 4.5. Western Blot Analysis of Proteins

Total intracellular protein, crude enzymes (40 µg) and pure enzymes (5 µg) were separated using 12% acrylamide gels with the addition of 4× Laemmli loading-buffer. Separated proteins were transferred onto a PVDF blotting membrane (GE Healthcare No. 1060022) and blotted in Tris-glycine pH 8.3 running buffer at 100 V for 45 min, after activation of the membrane in 96% ethanol for around 10 s. The membrane was blocked with 2% skim milk in 0.01 M TBS (Tris-based sodium chloride pH 7.6) buffer containing 0.1% Tween 20 (TBS_T buffer) for 60 min. The membrane was then incubated in TBS_T buffer with monoclonal anti-polyhistidine peroxidase conjugated antibody (Sigma-Aldrich, Steinheim, Germany) at 1:10.000 dilutions in a total volume of 30 mL for 1 h. The membrane was washed in TBS_T buffer 3 × 10 min and TBS with 0.1% Tween20 for 20 min. The bound antibodies were detected by horse radish peroxidase using the AEC Kit (Sigma-Aldrich, Steinheim, Germany) according to manufacturer’s protocol.

### 4.6. Carbohydrate–Polyacrylamide Gel Electrophoresis (C-PAGE)

Reaction mixtures containing 0.5 µg/µL enzyme solution in 20 mM Tris–HCl buffer pH 7.4, 250 mM NaCl and 10 mM CaCl_2_ (buffer A) and 1% weight/volume fucoidan in buffer A were incubated at 35 °C for 24–48 h. Each reaction mixture (10 µL) was mixed with 5 μL loading buffer (20% glycerol and 0.02% phenol red). Samples (5 μL) were electrophoresed at 400 V through a 20% (*w*/*v*) 1 mm thick resolving polyacrylamide gel with 100 mM Tris-borate buffer pH 8.3 for 1 h. Gel staining was performed in two steps, first with a solution containing 0.05% alcian blue 8GX (Panreac, Barcelona, Spain) in 2% acetic acid for 45 min and then with 0.01% O-toluidine blue (Sigma-Aldrich, Steinheim, Germany) in 50% aqueous ethanol and 1% acid acetic. The hydrolysate standard was obtained after enzymatic reaction of FFA2 on *Fucus evanescens* fucoidan [27].

### 4.7. SEC Analysis

High Performance Size Exclusion Chromatography was performed using an Ultimate iso-3100 SD pump with a WPS-3000 sampler (Dionex, Sunnyvale, CA, USA) connected to an RI-101 refractive index detector (Shodex, Showa Denko K.K., Tokyo, Japan). One hundred microliters of three times diluted reaction mixtures were loaded on a Shodex SB-806 HQ GPC column (300 × 8 mm) equipped with a Shodex SB-G guard column (50 mm × 6 mm) (Showa Denko K.K., Tokyo, Japan). Elution was performed with 100 mM sodium acetate pH 6 at a flow rate of 0.5 mL/min at room temperature. Pullunan standards were used as references.

## Figures and Tables

**Table 1:** Fucoidan-degrading enzymes, features, molecular weight and expression strains used.

Enzyme Name/GenBank No.	Organism	Features ^a^	Length (aa) ^b^	Expected MW (kDa)	*E. coli* Expression Strains
FcnA CAI47003.1	*Mariniflexile fucanivorans* SW5	nd	1007	nd	Nd
FcnA2	*Mariniflexile fucanivorans* SW5	His6 (N-term)	799	88	BL21 (DE3) pGro7 ^c^
FcnAΔ229	*Mariniflexile fucanivorans* SW5	His10 (N-term)	720	80	BL21 (DE3) pGro7 ^c^
Fda1 AAO00508.1	*Alteromonas* sp. SN-1009	His10 (N-term)	804	87	BL21 (DE3) pGro7 ^c^
Fda1Δ145	*Alteromonas* sp. SN-1009	His10 (N-term) and His10 (C-term)	669	73	BL21 (DE3) pGro7 ^c^
Fda1Δ395	*Alteromonas* sp. SN-1009	His10 (N-term) and His10 (C-term)	419	46	BL21 (DE3) pGro7 ^c^
Fda2 AAO00509.1	*Alteromonas* sp. SN-1009	His10 (N-term)	868	94	BL21 (DE3) pGro7 ^c^
Fda2-His	*Alteromonas* sp. SN-1009	His10 (N-term) and His10 (C-term)	878	95	BL21 (DE3) pGro7 ^c^
Fda2Δ146	*Alteromonas* sp. SN-1009	His10 (N-term) and His10 (C-term)	732	80	BL21 (DE3) pGro7 ^c^
Fda2Δ390	*Alteromonas* sp. SN-1009	His10 (N-term) and His10 (C-term)	488	53	BL21 (DE3) pGro7 ^c^
FdlA AAO00510.1	*Flavobacterium* sp. SA-0082	His10 (N-term)	684	74	C41 (DE3)
FdlB AAO00511.1	*Flavobacterium* sp. SA-0082	His10 (N-term)	692	76	C41 (DE3)

nd, not determined in this study. ^a^ Wild type signal peptide had been removed for codon-optimised synthesised construct; ^b^ Includes his-tags; ^c^ groES-groEL chaperone expressed from the pGro7 plasmid.

## Supplementary Materials

The following are available online at http://www.mdpi.com/1660-3397/16/11/422/s1, Figure S1: Recombinant expression of Fda1 in *E. coli*. (A) SDS-PAGE; and (B) Western blot of: (1) Autoinduced cells; (2) the cell debris (after sonication and protein extraction); and (3) crude extract after sonication and centrifugation. (St) is the protein plus molecular weight marker; Figure S2: Predicted protein domain structures of Fda1 and Fda2. Domains were predicted using NCBI conserved domain database (cdd) search tool and both proteins were found to contain two predicted LamG (Laminin G) superfamily domains. In Fda1, the domains span from position 429 to 574 aa and from 670 to 809 aa. For Fda2, the domains span from 496 to 641 aa and from 737 to 876 aa. Arrows indicate the points of truncation. Deletion mutants were named according to deletion from the C-terminal end, i.e., Fda1Δ145, Fda1Δ395, Fda2Δ146, and Fda2Δ390; Figure S3. (A) SDS-PAGE; and (B) Western blot of induced cells of: (1) Fda1; (2) Fda1Δ145; (3) Fda1Δ395; (4) Fda2-His; (5) Fda2; (6) Fda2Δ146; and (7) Fda2Δ390. St is the protein plus molecular weight marker (Bio-Rad Laboratories, Hercules, CA, USA). Table S1: Primers for constructing C-terminal deletion mutants.
