# Supplementary material for: Novel Enzyme Actions for Sulphated Galactofucan Depolymerisation and a New Engineering Strategy for Molecular Stabilisation of Fucoidan Degrading Enzymes
Source: Mar Drugs. 2018 Nov 1;16(11):422. doi: 10.3390/md16110422 (PMC6267234; doi:10.3390/md16110422)
Supplement: Supplementary file 1 [file marinedrugs-16-00422-s001.zip › marinedrugs-373457-SI.pdf]

## *Supplementary Material*

# Novel enzyme actions for sulfated galactofucan depolymerization and a new engineering strategy for molecular stabilization of fucoidan degrading enzymes

**Hang T.T. Cao[1,2], Maria D. Mikkelsen[1], Mateusz J. Lezyk[1], Ly M. Bui[2], Van T.T. Tran[2], Artem S. Silchenko[3], Mikhail I. Kusaykin[3], Thinh D. Pham [2], Bang H. Truong[2], Jesper Holck[1], and Anne S. Meyer[1,*]**

***Correspondence: asme@dtu.dk (A.M) Tel.: +45 45252598**

## 1 Supplementary Figures and Tables

### 1.1 Supplementary Figures

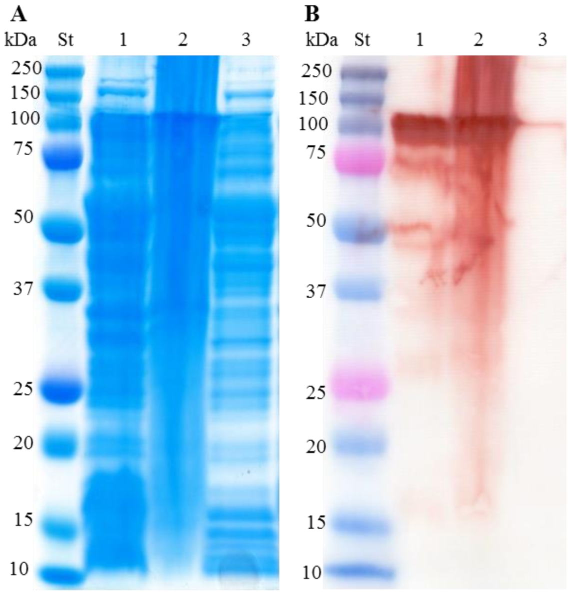


**Figure S1. Recombinant expression of Fda1 in *E. coli*.** A) SDS-PAGE and B) Western blot of 1) Autoinduced cells; 2) the cell debris (after sonication and protein extraction) and 3) crude extract after sonication and centrifugation. (St) is the protein plus molecular weight marker. The appearance of multiple bands in the cell debris Western blot analysis indicates that the tagged Fda1 degraded inside the cells; the intensity of the bands suggests that the recombinant enzyme was expressed, but that the expressed enzyme was not soluble, hence likely retained in inclusion bodies.

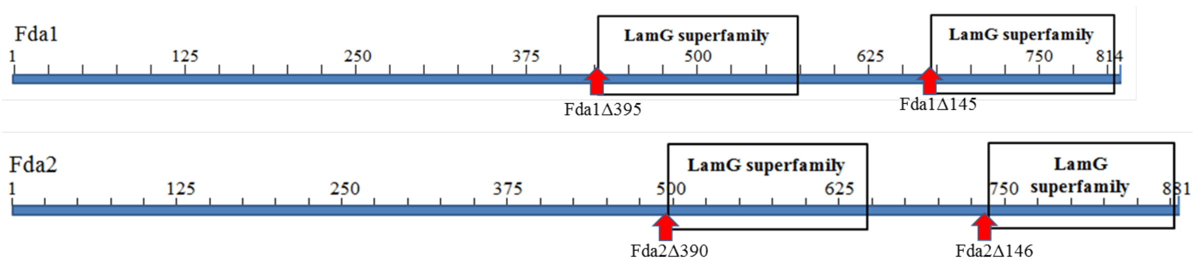


**Figure S2. Predicted protein domain structures of Fda1 and Fda2**. Domains were predicted using NCBI conserved domain database (cdd) search tool and both proteins were found to contain two predicted LamG (Laminin G) superfamily domains. In Fda1 the domains are spanning from position from 429 to 574aa and 670 to 809aa. For Fda2 the domains are spanning from 496 to 641aa and 737 to 876aa. Arrows indicate the points of truncation. Deletion mutants were named according to deletion from the C-terminal end, i.e. Fda1Δ145, Fda1Δ395, Fda2Δ146, and Fda2Δ390.

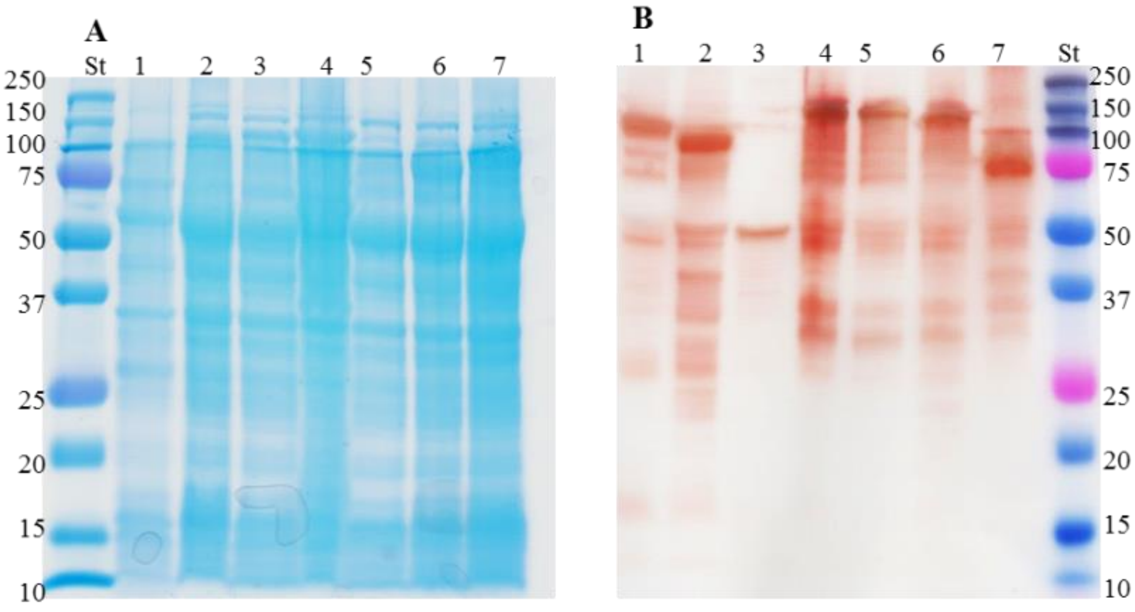


**Figure S3.** A) SDS-PAGE and B) western blot of induced cells of 1) Fda1; 2) Fda1Δ145; 3) Fda1Δ395; 4) Fda2-His, 5) Fda2; 6) Fda2Δ146; 7) Fda2Δ390. St is the protein plus molecular weight marker (Bio-Rad Laboratories, Hercules, CA, USA). Degradation is already evident before sonication

**Table S1: Primers for constructing C-terminal deletion mutants**

| Genes | Primers | Size (bp) |
|---|---|---|
| ***Fda1*** Forward primer: 5′- AGTCTACGGTCTCACATGGAAACCAAAGCAGATTGGATGCAG - 3′ | | **2423** |
| *Fda1Δ145* | Reverse primer : 5′- CAGTCATCTCGAGTTAATGGTGATGGTGATGATGGTGGTGATGAT GATAACCTTTACTATCTTTGCTGTTGTCGGTG -3′ | 1980 |
| *Fda1Δ395* | Reverse primer: 5′- CAGTCATCTCGAGTTAATGGTGATGGTGATGATGGTGGTGATGAT GTTTACCACGGGTAACATCATCAACAAAATTG -3′ | 1230 |
| **F*da2*** Forward primer: 5′- AGTCTACGGTCTCACATGAGCAGTCCGGAAGTTGAAGTTGATAATG - 3′ | | 2615 |
| *Fda2-His* | Reverse primer: 5′- AGTCTACGGTCTCACATGAGCAGTCCGGAAGTTGAAGTTGATAA TG - 3′ | 2615 |
| *Fda2Δ146* | Reverse primer: 5′- CAGTCATCTCGAGTTAATGGTGATGGTGATGATGGTGGTGATGAT GACCTTTGTTATCGCGTTTGTTGTTGG -3′ | 2169 |
| *Fda2Δ390* | Reverse primer: 5′- CAGTCATCTCGAGTTAATGGTGATGGTGATGATGGTGGT ATGATGTTCACGGGTCACATCATCAATAAAGG -3′ | 1437 |
